# Response: A commentary on: “Neural overlap in processing music and speech”

**DOI:** 10.3389/fnhum.2015.00491

**Published:** 2015-09-16

**Authors:** Barbara Tillmann, Emmanuel Bigand

**Affiliations:** ^1^Centre National de la Recherche Scientifique, UMR5292, INSERM, U1028, Lyon Neuroscience Research Center, Auditory Cognition and Psychoacoustics TeamLyon, France; ^2^University Lyon 1Lyon, France; ^3^Centre National de la Recherche Scientifique-LEAD, Université de BourgogneDijon, France; ^4^Institut Universitaire de FranceParis, France

**Keywords:** music and language, cognitive processes, sensory influences, interference, domain specificity

In comparison to a more classical approach investigating the modularity of music and language processing, recent research focuses on the investigation how and to what extent music and speech processing share neural correlates. This research has implications for the use of music for education and rehabilitation, and provides us with further insights regarding origins and evolution of music. As reviewed by Peretz et al. (2015), neuroimaging studies have been strongly contributing to this debate, suggesting both neural overlap and separability. In their commentary, Kunert and Slevc (2015) point out that behavioral and electrophysiological studies can also contribute to this investigation, and they provide an overview of research using a music-language interference paradigm.

In this paradigm, musical sequences and linguistic sentences were presented simultaneously. Each material (or both at the same time) can introduce a structural violation (or a more complex structure), and behavioral and electrophysiological measures are recorded to investigate whether the violation of the structure in one material (e.g., music) influences the processing of the structure in the other material (e.g., language). For example, participants read syntactic garden-path sentences, presented segment-by-segment and time-locked to the chords of a musical sequence (Slevc et al., 2009). These chords were either musically correct and expected (respecting musical syntactic-like structures), or incorrect and unexpected (i.e., an out-of-key chord). Results reveal interference of the musical material with the processing of linguistic syntax. Some studies have compared this interference effect with the effect of musical structures on semantic structure processing. The different result patterns have been interpreted in terms of interference being syntax-specific, pointing to more general structural integration or reflecting shared attention and cognitive control.

Comparing music and language processing, whether using neuroimaging, behavioral or electrophysiological methods, requires careful control and matching of the experimental material. First, when investigating the processing of cognitive structures and expectancy violations, care must be taken that the introduced structure violations (or manipulations) do not create additional violations, which might provide alternative explanations. Second, the manipulations in the material of the two domains need to be comparable in terms of their complexity (also when comparing syntactic and semantic processing).

The first point is particularly crucial for musical structure manipulations: the material must be constructed to exclude explanations based on low-level processing, which might provide a more parsimonious interpretation of the data than higher-level cognitive structure processing (e.g., Bigand et al., 2014; Collins et al., 2014). In Western tonal music, sensory and cognitive structures are indeed entwined, leading psychoacoustic and cognitive approaches to provide highly correlated accounts of musical structures (e.g., Bigand et al., 1996; Leman, 2000). Psychoacoustic approaches have challenged cognitive approaches that claimed for musical syntax processing: a short-term sensory memory model, operating on echoic images of periodicity pitch, can account for the musical functions of tones in tonal contexts (Leman, 2000).

This long-standing debate in music cognition research does not only concern the investigation of musical structure processing, but also the investigation of interference between musical and linguistic (syntactic, semantic) processing. This research domain should thus also question the relevance to use out-of-key violations as these are not only violating tonal structures and tonal expectations based on listeners' knowledge, but are also violating sensory expectations based on information stored in sensory memory buffer. These sensory violations compromise the unambiguous interpretation of interactive data patterns in terms of shared neural resources for musical and linguistic structure processing.

Eight of the ten studies listed in Kunert and Slevc (2015) used musical structure violations that introduced out-of-key notes or chords. Consequently, the question rises in how far the observed interference and interactive patterns are due to the sensory violations of the out-of-key events rather than musical syntax processing. Some of the authors were aware of potential alternative influences of other “types of musical unexpectancy,” which might attract attention, and used control conditions that introduced a timbre or loudness change (Fedorenko et al., 2009; Slevc et al., 2009; Fiveash and Pammer, 2014). However, it seems difficult to match changes on timbre or loudness dimensions in terms of the degree of violation to changes due to an out-of-key event. It might be that the violation of sensory expectations is stronger for out-of-key events, and/or that the out-of-key event combines sensory and cognitive violations, leading to a stronger violation.

This discussion leads to the second point, notably the comparability of the structural complexity and violations across music and language materials as well as within the language material, such as the comparability of syntactic and semantic expectancy violations when investigating their interactions with musical expectancy violations. For example, semantic violations based on correct, but low-cloze probability words might be less strong than syntactic violations based on syntactic errors (gender violations) or syntactic complex sentences, thus being less strongly subjected to interference with musical violations (Hoch et al., 2011; Perruchet and Poulin-Charronnat, 2013).

Where to go from here? Research investigating neural correlates as well as interference patterns between music and language processing should take into account debates and advances of music cognition and psycholinguistic domains: the need to disentangle musical structure violations from sensory violations (e.g., Leman, 2000; Bigand et al., 2014) as well as the need to equalize strength of structure manipulations across linguistic dimensions (e.g., syntax and semantics; Gibson and Fedorenko, 2013) and between musical and linguistic dimensions. Using other materials might complement the investigation of the interference with musical structures, such as arithmetic processing that allows manipulating more directly the degree of complexity of the structures (e.g., Hoch and Tillmann, 2012). Assuring equal strengths of manipulations across dimensions requires additional testing, including baseline conditions (without the concurrent manipulation of the other dimension), as done similarly in studies using Garner's interference paradigm (Garner, 1974). Even though initially developed to investigate perceptual processes, Garner's paradigm has been used to study sensory and linguistic processes (e.g., Melara and Marks, 1990) or text and melody in song (see Lidji, 2007). It also calls the domain to further study the directionality of the interference between music and language processing (with most studies having investigated the effect of music on language processing, see however Steinbeis and Koelsch, 2008).

## Conflict of interest statement

The authors declare that the research was conducted in the absence of any commercial or financial relationships that could be construed as a potential conflict of interest.
